# Driving Performance Evaluation of Shuttle Buses: A Case Study of Hong Kong–Zhuhai–Macau Bridge

**DOI:** 10.3390/ijerph19031408

**Published:** 2022-01-27

**Authors:** Ming Lv, Xiaojun Shao, Chimou Li, Feng Chen

**Affiliations:** 1 CCCC Wenshan Highway Construction & Development Co., Ltd., Wenshan 663099, China; lvming93@outlook.com (M.L.); lichimou@outlook.com (C.L.); 2 The Key Laboratory of Road and Traffic Engineering, Ministry of Education Tongji University, Shanghai 201804, China; fengchen@tongji.edu.cn

**Keywords:** risky driving behaviour, random parameter, negative binomial regression

## Abstract

The risky behaviours of bus drivers are of great concern to public health and environmental sustainability, especially for the buses operated between cities. With this in mind, the present study examined the distribution of risky behaviours among bus drivers, and the contributing factors to risky performance. To achieve this, 1648 records of GPS trajectory data and 8281 records of advance warning message data from Hong Kong–Zhuhai–Macau Bridge shuttle buses were obtained. The temporal and spatial distribution of risky behaviours was analysed. A random parameters negative binomial model was developed to further investigate the relationship between speed-related factors and risky behaviours. The results indicated that the warning of safety distance, lane departure, forward collision, and distraction were more likely to occur on weekdays. The period between 14 and 16 o’clock obtained the highest frequency of safety distance and lane departure warnings. Regarding the model estimation results, indicators reflecting average speed, acceleration, and number of trips per day showed a statistically significant impact on safety distance and lane departure warnings. Also, the acceleration of bus drivers showed a mixed impact on lane departure warnings. Corresponding implications were discussed according to the findings to reduce the frequency of risky behaviours in shuttle bus operations.

## 1. Introduction

According to the road safety report issued by World Health Organization, the number of road traffic deaths has reached 1.35 million each year, leading to great concerns on public health and environmental sustainability [1]. To mitigate the circumstances, efforts have been put forth to encourage the use of public transport to improve road safety by reducing vehicular traffic [2]. However, there is growing scientific evidence that professional drivers experience more accidents than non-professional drivers [3,4]. Professional drivers are facing more and more work stress, which, in turn, results in risky driving behaviours, such as lane departure, fatigue, and distraction [5]. Risky driving behaviours can be classified into two categories: errors and violations [6]. Errors are defined as the failure of the intended action to achieve the desired goals (e.g., misjudging the road conditions, distracted by passengers). Violations, on the other hand, refer to the intended action to break the traffic rules (e.g., exceed the speed limit). Hence, it is important to identify the hidden danger and contributing factors to the risky behaviour of public transport drivers.

This is especially the case with bus drivers. Unlike private transport, the traffic accidents of buses are often severe, owing to its unique attributes of weight, height, length, number of passengers, and braking performance. Taking Hong Kong as an example, there is a notable increase in traffic accident involvement rate (number of vehicles involved per million vehicle-kilometres) for public buses since 2009 [7]. Of the 25,726 traffic accident-involved vehicles in 2018, public buses (including public light buses) constituted the third-largest share (14.60%) [7].

Previous studies on risky behaviours of bus drivers seek to understand their relationship with various factors in terms of psychologies [8], personalities [9,10], performance [11], and environmental conditions [12]. Among these studies, self-reported questionnaires were the commonly adopted method in collecting risky-behaviours-related data. For instance, Useche et al. [8] examined the self-reported risky driving behaviour based on 524 male drivers of bus rapid transit, and found that risky driving behaviour could be predicted from stress-related work conditions. Mallia et al. [9] conducted questionnaires of 301 bus drivers, and found that risky behaviour was directly associated with personality traits such as altruism and excitement-seeking. However, the use of self-reported measures suffers from social desirability bias, in which respondents tend to provide a more positive answer than their actual thought to make them look better in the eyes of others [13]. In particular, this kind of method might inflate the relationship between risky behaviours and contributing factors.

As an alternative, the use of observed behavioural data and official crash records were suggested [14]. Krajewski et al. [15] identified driving fatigue from steering wheel movement. Choi et al. [16] analysed and classified driving behaviour based on in-vehicle sensors. Carmona et al. [17] presented a driving behaviour identification method by using onboard information and advanced embedded sensors. Also, a substantial number of researchers have explored the contributing factors to bus-related crashes via official crash records [11,12,18,19,20]. It was clear that real-time data provided a powerful way of analysing driving behaviours. However, limited studies have been conducted on examining the risky behaviour via observed behavioural data for bus drivers [9]. Hickman and Hanowski [21] analysed the impact of distracted driving (e.g., talking on a cell phone, texting) on crash risk by using naturalistic driving data collected from bus drivers. Eyes off the forward road behaviours were found to significantly increase the involvement rate in a safety-critical event. Jacob and Anjaneyulu [22] investigated the relationship between road geometry and operating speed for bus drivers. It was found that the speed was more sensitive to curve length than curve radius. 

Although previous studies have made great contributions to before-crash manoeuvres and crash injury severity of buses, few of these studies have focused on identifying the driving performance and risky behaviour of bus drivers; especially for the buses operated between cities, where the driving speeds are relatively higher compared to urban buses. The driving safety of such buses is of great concern to both passengers and operators. Therefore, it is necessary to evaluate the driving performance of shuttle bus drivers.

Based on the aforementioned discussion, this study aimed to understand the distribution of risky behaviours among bus drivers, and identify the potential relationship between speed-related factors and risky behaviours. Using the GPS data and the advance warning message data from Hong Kong–Zhuhai–Macau Bridge (HZMB) shuttle buses, this study first analysed the temporal and spatial distribution of the risky behaviour of bus drivers, then developed random parameters negative binomial models to provide a deep understanding of contributing factors to risky behaviours, such as driving close to the front vehicle, and lane departure. This model is statistically superior to the fixed parameters negative binomial approach, as it captures the possible unobserved factors [23,24]. The findings of this study will shed light on bus operators to better understand their risky behaviour, and, therefore, allow special training in driving behaviour to provide a better and safer service.

## 2. Data Description

**Figure 1:**
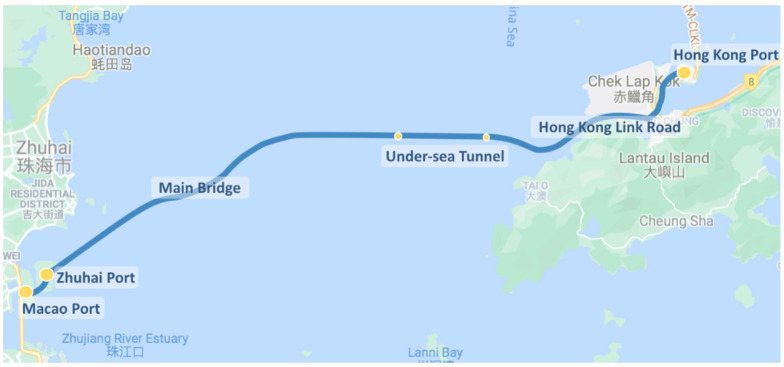
The route of the Hong Kong–Zhuhai–Macau Bridge shuttle bus.

The data introduced in this study were collected from HZMB shuttle buses, which operated non-stop on the longest bridge-cum-tunnel sea crossing in the world. The route length was approximately 40 km from Hong Kong port to Zhuhai port and Macau port. The bus routes, with essential locations, are illustrated in Figure 1. 

Data quality determines the validity of findings [25]. Four months of data between 1 October 2020 and 31 January 2021 were collected via a GPS data logger device, Advanced Driver-Assistance System (ADAS), and facial recognition system. The GPS device tracks temporal and spatial data. Mobileye-powered ADAS sensors were mounted below the windshield outside the bus to detect alarming events ahead of the vehicle. A Hikvision facial recognition camera was mounted above the dashboard inside the bus to monitor any risky behaviour of drivers. The original dataset consisted of 204 vehicles, with more than 10 million records. All the drivers were male drivers.

### 2.1. GPS Data

GPS data were measured every 5 s, including the UTC time, latitude, longitude, altitude, direction, instantaneous speed, number of satellites in view, and position dilution of precision (PDOP). To obtain a reliable database, the records where the number of satellites was less than 4, or PDOP greater than 7, were discarded. Also, static vehicles and vehicles operated outside the bus route were excluded. After data screening, the remaining 1,136,229 records (including 115 vehicles) were grouped by number plate and date.

Finally, a total of 1648 records were obtained from the four-month GPS database to investigate the driving behaviour of shuttle buses. Each record represented the trips per vehicle per day. Note that GPS data at the undersea tunnel and Scenic Hill tunnel were not included in this study, as there was no reliable signal for the GPS device to measure.

### 2.2. Advance Warning Message Data

There were seven types of advance warning messages detected by ADAS (safety distance, lane departure, forward collision, and pedestrian collision) and the facial recognition system (distraction, calling, and fatigue). The corresponding definitions of each warning message are shown in Table 1. After filtering the false positive warnings, a total of 8281 records were selected for further analysis. Each record represented a warning message of a bus with a date and an approximate location. Note that due to technical issues, only data from January 2021 (1887 records) were recorded with time stamps and durations. The remaining data were recorded with date only.

Among all the warning messages, 91.8% were generated from ADAS, and the rest from the facial recognition system. In particular, safety distance accounted for the most (64.70%). Next, was lane departure (19.93%), distraction (7.37%), and forward collision (6.88%). There were minor records for calling, pedestrian collision, and fatigue, which accounted for 0.66%, 0.29%, and 0.17%, respectively.

### 2.3. Variables Selection for Model Estimation

Based on the above dataset, speed-related and acceleration-related variables were selected in analysing the contributing factors to the risky behaviours of shuttle bus drivers. To check for possible collinearity, Pearson’s correlation tests were conducted. Two pairs were found to be correlated, with a correlation coefficient of −0.82 between mean acceleration and mean deceleration, and 0.62 between the standard deviation of acceleration and standard deviation of deceleration. Hence, acceleration manoeuvre was selected for further analysis. Note that there was no sudden acceleration (greater than 3 m/s^2^) or sudden deceleration (less than −3 m/s^2^) captured during the bus operation. 

As a consequence, six variables were tested in models for this study, including mean and standard deviation of speed, the mean and standard deviation of acceleration, number of times speeding per day, and number of trips per day. A summary of the statistics of each variable is presented in Table 2.

## 3. Methodology

This study focused on identifying the temporal and spatial distribution of the risky behaviour of shuttle buses, as well as the potential relationship between driving-speed-related variables and risky behaviours.

Temporal characteristics referred to the daily and hourly distributions of different types of warning messages across all shuttle buses. Spatial characteristics were identified using the reported location of warning messages. To identify the contributing factors to risky behaviours, count data models were used in this study. As two categories of the most frequently used approach, a Poisson model and negative binomial model have been commonly applied in count data modelling [23,26,27]. Poisson distribution is relatively easy to interpret. However, it follows a restrictive assumption that the variance of the count variable equals its mean [28]. Since the data of risky behaviours in the current study were over-dispersed, negative binomial regression was introduced. Negative binomial distribution has been proved to better represent count data than Poisson distribution [29].

The negative binomial regression model is derived by introducing the probability of $y_{i}$ as the risky behaviour counts of each observation i, which is defined by Equation (1) [30]:

(1) $$P\left(y_{i}\right)=\frac{\Gamma \left[\left(1/\alpha \right)+y_{i}\right]\;}{\Gamma \left(1/\alpha \right)y_{i}!}{\left(\frac{1/\alpha }{\left(1/\alpha \right)+\lambda_{i}}\right)}^{1/\alpha }{\left(\frac{\lambda_{i}}{\left(1/\alpha \right)+\lambda_{i}}\right)}^{y_{i}}$$

where α is the over-dispersion parameter, Γ(·) is a Gamma function, and $\lambda_{i}$ is given by Equation (2):

(2) $$\lambda_{i}=EXP\left(\beta X_{i}+\varepsilon_{i}\right)$$

where $X_{i}$ is a vector of independent variables, β is a vector of estimable parameters, and $EXP\left(\varepsilon_{i}\right)$ is a Gamma-distributed disturbance term with mean one and variance α. 

However, this standard negative binomial model might lead to potential bias by treating the parameters β as constant across observations, which restricts each variable to have the same effect on every individual observation [31]. Hence, to account for the influence of unobserved heterogeneity, a random parameter negative binomial model is derived by adding a randomly distributed error term $\varphi_{i}$ (e.g., a normal distribution with mean zero and variance $\sigma^{2}$) [32,33,34], as shown in Equation (3):

(3) $$\beta_{i}=\beta +\varphi_{i}$$

The log-likelihood with this random parameter can be expressed in Equation (4):

(4) $$LL=\underset{\forall i}{\sum }ln\int_{\varphi_{i}}g\left(\varphi_{i}\right)P\left(n_{i}|\varphi_{i}\right)d\varphi_{i}$$

where g(·) is the probability density function of $\varphi_{i}$.

Since the interpretation of the parameter estimation results was not straightforward, marginal effects were calculated to determine the impact of one unit change in an independent variable on the probability of the frequency of risky behaviours; computed as Equation (5) [30]:

(5) $$ME_{x_{ik}}^{\lambda_{i}}=\frac{\partial \lambda_{i}}{\partial x_{ik}}=\beta_{k}EXP\left(\beta X_{i}\right)$$

where $x_{ik}$ is the value of the kth independent variable for observation i, $\beta_{k}$ refers to the estimated parameter for the kth independent variable.

Furthermore, to compare the differences between the random parameters model and their fixed parameters model, the likelihood ratio test was adopted, and can be defined as Equation (6) [30]:

(6) $${Χ}^{2}=-2\left[LL\left(\beta_{fixed}\right)-LL\left(\beta_{random}\right)\right]$$

where $LL\left(\beta_{fixed}\right)$ and $LL\left(\beta_{random}\right)$ are the log-likelihoods at the convergence of fixed parameters negative binomial model and random parameters negative binomial model, respectively. The ${Χ}^{2}$ statistic is a $\chi^{2}$ distribution with the degrees of freedom equal to the difference between the numbers of parameters of two models.

## 4. Results and Discussion

### 4.1. Temporal and Spatial Characteristics of Risky Behaviours

#### 4.1.1. ADAS Warnings

**Figure 2:**
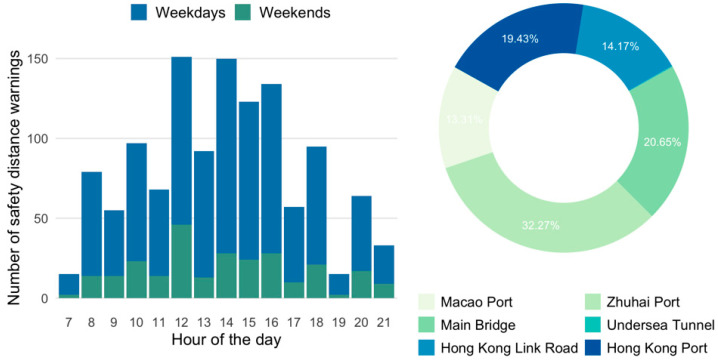
Temporal and spatial characteristics of safety distance warning. Note that in the doughnut chart Undersea Tunnel accounts for 0.17% of the warnings.

The daily, hourly, and spatial distributions of safety distance warnings are shown in Figure 2. As can be seen, the number of safety distance warnings on weekends (132.5 times per day) was much lower than on weekdays (192.6 times per day). This might be linked to the higher traffic density of roadways on weekdays, in which the distance between vehicles was shorter under congestion. Similar findings have been observed in previous studies that weekday crashes were more likely to happen in congested situations [35]. As for the hourly distribution, safety distance warnings were mostly detected at 12 o’clock and 14 to 16 o’clock, with more than 120 times of warnings per day for 51 buses. In other words, there were at least two warnings of safety distance for each shuttle bus driver in these periods. This could be explained as daily sleepiness, where drivers were prone to feel drowsy in the early afternoon [36]; therefore, reducing their ability to keep a safe distance to front vehicles. For the spatial distribution, 65.01% of safety distance warnings were detected near Zhuhai, Hong Kong, and Macao ports, where traffic flow was relatively high, especially at Zhuhai port. The rest of the warnings were detected at Hong Kong–Zhuhai–Macao Bridge, with most warnings occurring at the main bridge section. Few warnings were detected at the undersea tunnel. 

**Figure 3:**
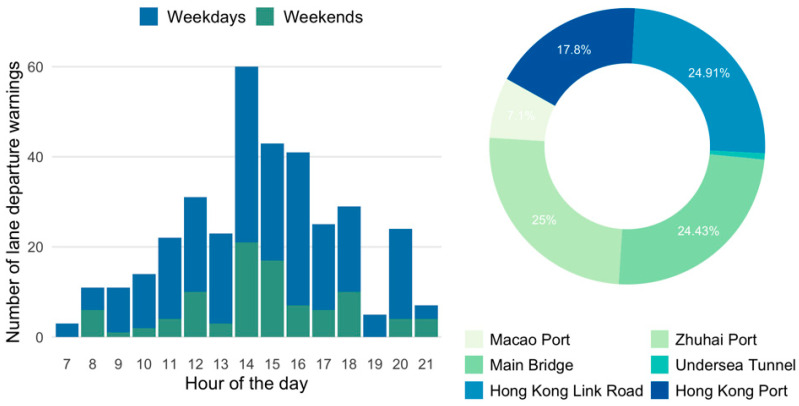
Temporal and spatial characteristics of lane departure.

As shown in Figure 3, the temporal distributions of lane departure warnings were similar to safety distance warnings. The average number of lane departure warnings on weekdays (50.8 times per day) was slightly higher than on weekends (47.5 times per day). It is possible that drivers are prone to improper lane changing behaviour during congestion. Further investigations are needed to find out the reason behind this. In line with the drivers’ drowsiness pattern [36], the period between 14 and 16 o’clock is associated with the highest frequency for drivers to drive across the lane. Special concerns should be paid in those periods to remind drivers of lateral and longitudinal control of buses. A short nap before driving, or an audio warning on board, might be useful in reducing such behaviours. The doughnut chart in Figure 3 illustrates the spatial distribution of lane departure warnings. As can be seen, the locations were evenly distributed among Zhuhai port, the main bridge, and Hong Kong Link Road. These locations accounted for nearly 75% of overall lane departure warnings. The rest of the warnings took place at the other two ports. Similarly, there were still fewer warnings of lane departure detected at the undersea tunnel.

**Figure 4:**
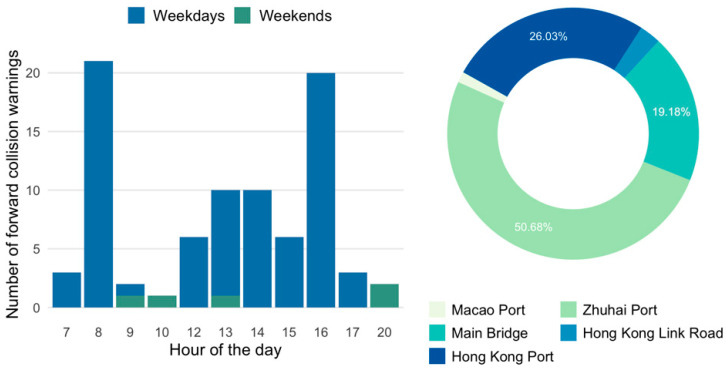
Temporal and spatial characteristics of forward collisions.

The temporal and spatial distribution of the forward collisions showed a different trend (Figure 4). More than 90% of the warnings were detected on weekdays. Also, the frequency of the forward collision warning was extremely high at 8 o’clock and 16 o’clock. This strong hourly pattern might be related to the fact that 8 and 16 o’clock at weekdays are the peak hours at Zhuhai and Hong Kong ports. Although previous studies have shown that forward collision warning had a great potential for preventing crash injuries [37], shuttle bus operators should still be alert to these periods to reduce the potential risks. For the spatial distribution, a majority of warnings took place at ports, with only 21.92% of warnings detected on the bridge. According to the onsite photos that the ADAS system captured during collision, most of the warnings detected near ports could be caused by kerb scratch while turning. Instead, the warnings detected on the bridge should be taken seriously, as the driving speed on the bridge was usually high (40~80 km/h). Any forward collision could be a great hazard to passenger and driver safety.

Due to the regulations that pedestrians are not allowed to walk on the bridge, all pedestrian collision warnings were detected at ports. However, it was hard to find any temporal distributions, as there were not enough observations.

#### 4.1.2. Facial Recognition Warnings

**Figure 5:**
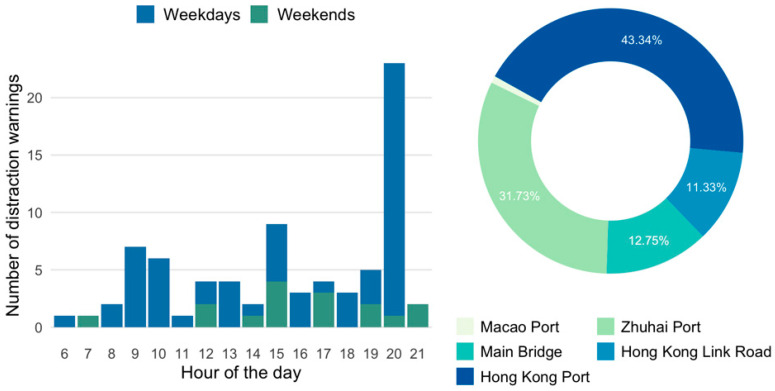
Temporal and spatial characteristics of distraction.

The facial recognition system detected the driver’s distraction, fatigue, and calling behaviours according to the eye and head movement. Eye movement here refers to the fluctuation of pupil diameter, as well as the percentage of eye closure over a period of time (PERCLOS). These indicators have been proved by many researchers to be an effective way in monitoring distraction and driving fatigue [38,39,40]. Similarly, head movement refers to the specific pose and position of head [41,42]. The temporal and spatial characteristics of distraction are illustrated in Figure 5. As can be seen, a majority of the warnings were detected on weekdays (12.2 times per day on weekdays, 8 times per day on weekends). Past studies have shown that driver distraction was affected by driving tasks, such as traffic and road conditions [43]. The heavy road traffic on weekdays could be one of the reasons. Moreover, it was found in the present study that drivers were more likely to be distracted at 20 o’clock. By deep-looking into the data, it can be seen that almost 80% of the distraction warnings at this specific hour were triggered by the same driver on a single trip. A training program on safe driving should be delivered to this driver. Among all the distraction warnings, more than 75% of them took place at ports, where drivers’ attentions were often attracted by other buses or staff at terminal ports. Despite the low percentage, the implication of distracted driving at the bridge could be serious, as the driving speed was normally fast. Recognising these trends, bus operators should pay more attention to roadways with high speed limits (the main bridge in this case study) to reduce the crash risk.

Due to limited observations of fatigue and calling, the temporal distributions of these warnings were not clear. However, it should be noticed that all the detected fatigue took place at the main bridge, whereas all calling warnings were detected at Hong Kong port.

### 4.2. Contributing Factors to Risky Behaviours

Due to insufficient observations, only two of seven warning messages were selected for further analysis. The results of likelihood ratio tests between fixed and random parameters models for each dataset are presented in Table 3. With all the p values below 0.001, we are 99.99% confident that the random parameters negative binomial models outperformed the corresponding fixed ones. Hence, the following results and discussion were based on the random parameters models.

**Figure 6:**
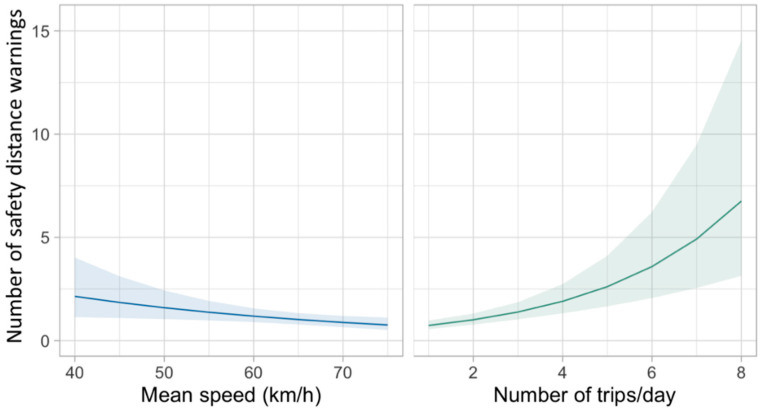
Marginal effects of safety distance warnings.

**Figure 7:**
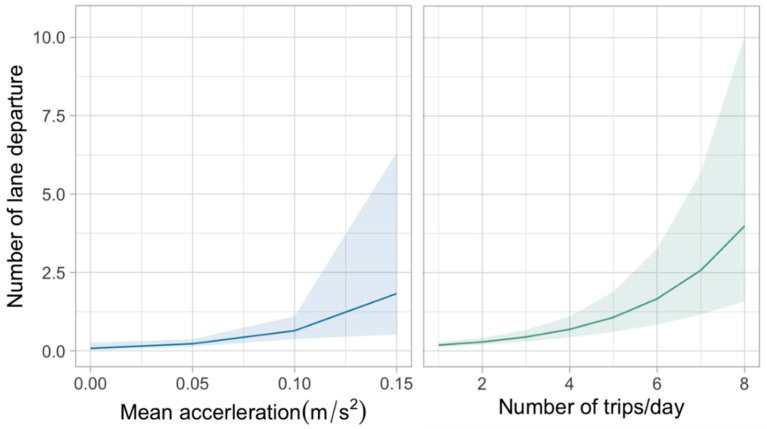
Marginal effects of lane departure warnings.

Random parameters model estimation results for safety distance warnings and lane departure warnings are presented in Table 4 and Table 5, respectively. The marginal effects for these two datasets are shown in Figure 6 and Figure 7.

#### 4.2.1. Safety Distance Variables

Regarding the model estimation results, Table 3 shows that two variables have significant impacts on safety distance warnings. In particular, the probability of safety distance warnings slightly increased with lower speed. However, it does not show that safety distance warnings decreased with higher speed. As discussed in Section 4.1.1, more than 60% of these warnings were detected near ports, where drivers were either at their departure or reaching their destination. In other words, the velocity at ports was relatively lower than that on the bridge. Meanwhile, the traffic density was relatively higher. Therefore, given these circumstances, it was reasonable to get the present results. 

Another variable that showed significant impacts was the number of trips, which is positively associated with safety distance warnings. As illustrated in Figure 6, the number of safety distance warnings increased greatly with the number of trips. For bus drivers who took eight trips per day, the average number of safety distance warnings increased to almost one warning per trip. One possible explanation was that drivers’ fatigue accumulated with the number of trips, and, therefore, it was getting hard to keep a safe distance with front vehicles. However, there were not enough observations of fatigue warnings to support this. Hence, further investigation with adequate fatigue observations is needed to obtain a better understanding.

#### 4.2.2. Lane Departure Variables

The model results shown in Table 4 suggest that two variables were statistically associated with lane departure warnings. One variable, the number of trips, was found to have a similar impact on lane departure as those in safety distance warnings. Another variable, mean acceleration, was found to be statistically significant only in determining lane departure warnings. To avoid repetition, this section will only discuss the latter one.

Specifically, the possibility of lane departure increased slightly when accelerating. It is possible that some of the detected lane departure warnings were actually lane changing behaviours. As has been studied before, most drivers accelerate before lane change [44]. The only difference was that the drivers forgot to activate turn signals. Hence, regulations on the driving behaviour of bus drivers when lane changing could be enhanced. However, the effect seemed to be mixed, as the mean acceleration was found to have a random effect on lane departure behaviour. It implied a 64.35% of observations (above zero) were more likely to reduce the lane keeping ability at higher accelerations, whereas 35.65% of observations (below zero) were found to decrease the lane keeping ability at lower accelerations. Overall, some of the drivers showed an unintentional lane departure while accelerating, but the impacts of acceleration on lane keeping varied across individuals.

There were, of course, limitations to the present study. One was the limited dataset collected from HZMB shuttle buses. Due to the impact of COVID-19, the operation schedule of shuttle buses was altered, resulting in a limited operation frequency. The other was the incomplete records of some observations, in which the analysis of hourly distribution of risky behaviour was based on a one-month dataset, only due to the incomplete sampling of time stamps. Future studies with more observations are needed to gain a deep understanding of the driving performance of shuttle buses.

## 5. Conclusions

The risky behaviours of bus drivers are of great concern to public health and environmental sustainability. This study analysed the daily, hourly, and spatial distribution of risky driving behaviours based on the advance warning messages collected by HZMB shuttle buses. Moreover, along with GPS data, the random parameters negative binomial models were developed to further investigate the relationship between speed-related factors and risky behaviours. The likelihood ratio tests were conducted to evaluate the goodness of fit between fixed parameters negative binomial models and their random parameters counterparts. The model estimation results demonstrated the random parameters negative binomial models outperformed the corresponding fixed one. Based on the results from temporal and spatial distribution, as well as the model estimation results, the main findings were concluded as follows:

1. The risky behaviours, including driving close to the front vehicle, lane departure, forward collision, and distraction, were more likely to occur on weekdays. Moreover, driving between 14 and 16 o’clock was the period most likely to receive safety distance and lane departure warnings.
2. Considering the spatial characteristics, despite the fact that most of the risky behaviours were detected near Zhuhai, Hong Kong, or Macao ports, the warnings that occurred at Hong Kong–Zhuhai–Macao Bridge should be taken seriously, as the driving speed is normally high.
3. The impact of acceleration on lane departure was mixed, with 64.35% of shuttle bus drivers more likely to reduce their lane-keeping ability at higher accelerations, whereas 35.65% of drivers were found to decrease their lane-keeping ability at lower accelerations. Also, the number of trips within a day for shuttle bus drivers was positively associated with safety distance and lane departure warnings.

Based on the findings, a number of practical implications could be developed. First, according to the temporal and spatial distributions of risky behaviours, more attentions are needed by bus operators and safety agencies in those particular periods and locations. Second, to reduce the risky behaviour of lane departure and driving too close to the front vehicle, safety and enforcement agencies should enhance the speed regulations on bus drivers. These implications are not unique to HZMB buses. It could also be extrapolated to bus drivers who operate on crosstown roads or Bus Rapid Transit systems, as they share similar features (long distance between stops, fast and reliable operating speed).

## Figures and Tables

**Table 1:** Definitions of advance warning messages.

Warning Messages	Definition	Records
Safety distance	Driving too close to the front vehicle.	5358
Lane departure	Moved out its lane without the turn signal on.	1650
Forward collision	Impending collision with front vehicles or obstacles.	570
Pedestrian collision	Impending collision with pedestrians.	24
Distraction	Driver’s head turned away from the road ahead.	610
Calling	Answering cell phone calls while driving.	55
Fatigue	Drowsy behaviour, such as yawning and slow eye closures.	14

**Table 2:** Statistics of variables.

Variables	Mean	Std. Dev.	Minimum	Maximum
Mean speed (km/h)	64.23	5.06	42.66	74.28
Std. dev. speed	14.897	3.25	3.857	24.792
Mean acceleration (m/s^2^)	0.064	0.018	0.019	0.133
Std. dev. acceleration	0.099	0.023	0.019	0.197
No. of speeding/day	1.427	8.083	0	130
No. of trips/day	2.112	1.175	1	8

**Table 3:** Likelihood ratio tests between fixed and random parameters models.

Dataset	Safety Distance	Lane Departure
$\chi^{2}$ value	24.72	93.55
Degrees of freedom	9	11
*p* value	<0.001	<0.001

**Table 4:** Model estimation results for safety distance warnings.

Variable	Random Parameters Negative Binomial Model	
	Coefficient	z Value
Constant	0.647	0.737
Standard deviation of parameter density function	0.750 **	
Mean speed	−0.030 **	−2.389
Standard deviation of speed	0.023	1.133
Mean acceleration	7.009	1.242
Standard deviation of acceleration	−1.573	−0.448
Number of speeding	0.004	0.494
Number of trips	0.317 ***	5.326
Number of observations	1647	
Log-likelihood at convergence	−2444.1	
Akaike Information Criterion (AIC)	4906.1	

Note: ***, ** refer to significance at 1%, 5% level.

**Table 5:** Model estimation results for lane departure warnings.

Variable	Random Parameters Negative Binomial Model	
	Coefficient	z Value
Constant	−3.298 ***	−2.835
Mean speed	0.001	0.067
Standard deviation of speed	−0.013	−0.538
Mean acceleration	20.886 ***	2.639
Standard deviation of parameter density function	13.321 ***	
Standard deviation of acceleration	−0.265	−0.061
Number of speeding	0.006	0.683
Number of trips	0.437 ***	6.065
Number of observations	1647	
Log-likelihood at convergence	−1508.6	
Akaike Information Criterion (AIC)	3039.1	

Note: *** refers to significance at 1% level.

## Data Availability

Restrictions apply to the availability of these data. Data was obtained from Hong Kong-Zhuhai-Macao Bridge Shuttle Bus Company Limited and are available from Feng Chen with the permission of Hong Kong–Zhuhai–Macao Bridge Shuttle Bus Company Limited.
